# Risk stratification in acute variceal bleeding: Far from an ideal score

**DOI:** 10.6061/clinics/2021/e2921

**Published:** 2021-06-23

**Authors:** Carla Luiza de Souza Aluizio, Ciro Garcia Montes, Glaucia Fernanda Soares Ruppert Reis, Cristiane Kibune Nagasako

**Affiliations:** Divisao de Gastroenterologia, Departamento de Clinica Medica, Faculdade de Ciencias Medicas, Universidade Estadual de Campinas (FCM/UNICAMP), Campinas, SP, BR

**Keywords:** Upper Gastrointestinal Bleeding, Variceal Bleeding, Cirrhosis, Risk Stratification

## Abstract

**OBJECTIVES:**

Acute variceal bleeding (AVB) results from rupture of esophageal or gastric varices. It is a life-threatening complication of portal hypertension. Nevertheless, it remains unclear how to predict adverse outcomes and identify high-risk patients. In variceal hemorrhage, high Child-Turcotte-Pugh (Child) and Model for End-stage Liver Disease (MELD) scores are associated with a worse prognosis. The Rockall system (Rockall), Glasgow-Blatchford (Blatchford), and AIMS65 scores have been validated for risk stratification for nonvariceal upper gastrointestinal bleeding; however, their use is controversial in AVB. The aim of this study was to compare the performance of Child, MELD, Rockall, Blatchford, and AIMS65 scores in risk stratification for rebleeding and/or mortality associated with AVB.

**METHODS:**

This retrospective study was conducted at a tertiary care hospital over 42 months. The outcomes were 6-week rebleeding and mortality. The AUROC was calculated for each score (1-0.9, 0.9-0.8, and 0.8-0.7, indicating excellent, good, and acceptable predictive power, respectively).

**RESULTS:**

In total, 222 patients were included. Six-week rebleeding and mortality rates were 14% and 18.5%, respectively. No score was useful for discriminating patients at a higher risk of rebleeding. The AUROCs were 0.59, 0.57, 0.61, 0.63, and 0.56 for Rockall, Blatchford, AIMS65, Child, and MELD scores, respectively. Prediction of 6-week mortality based on Rockall (AUROC 0.65), Blatchford (AUROC=0.60), and AIMS65 (AUROC=0.67) scores were also not considered acceptable. The AUROCs for predicting mortality were acceptable for Child and MELD scores (0.72 and 0.74, respectively).

**CONCLUSION:**

Rockall, Blatchford, and AIMS65 scores are not useful for predicting 6-week rebleeding or mortality in patients with AVB. Child and MELD scores can identify patients at higher risk for 6-week mortality but not for 6-week rebleeding.

## INTRODUCTION

Acute variceal bleeding (AVB) results from the rupture of esophageal or gastric varices and is a life-threatening complication of portal hypertension (PH) (1). Despite endoscopic and pharmacological innovations in recent decades, AVB is still associated with mortality rates >20% (2-6).

Although international guidelines (Baveno VI, AASLD, BSG) (7-9) recommend risk stratification of patients with AVB, it remains unclear how to predict adverse outcomes and identify high-risk patients.

Child-Turcotte-Pugh (Child) and Model for End-stage Liver Disease (MELD) scores were used to assess the severity of liver disease. Although these scores can be useful in predicting outcomes in patients with AVB (10), they have some limitations. Clinical variables in Child are subjective and can be influenced by the use of diuretics and lactulose. Both scores consider the international normalized ratio (INR); however, this isolated measure is not a reliable indicator of coagulopathy and liver function in patients with liver cirrhosis. In addition, there is variation in the INR values among different laboratories (11).

Several scoring systems have been developed to predict adverse outcomes in patients with nonvariceal upper gastrointestinal bleeding (UGIB). The Glasgow-Blatchford (Blatchford) and AIMS65 scores identify high-risk patients who need early upper gastrointestinal (GI) endoscopy and hospitalization, and low-risk patients who could be managed as outpatients with routine endoscopy (12). The Rockall system (Rockall) is a score designed to estimate the risk of rebleeding or death in patients with UGIB before and after endoscopy (12-14).

Data on the applicability of Rockall, Blatchford, and AIMS65 scores in AVB are conflicting. Some authors concluded that these scores are not useful for predicting outcomes in patients with AVB (15,16). In contrast, others have shown that MELD and AIMS65 scores can predict mortality, while Blatchford and Rockall scores can predict rebleeding (17).

Risk stratification identifies high-risk patients whose survival may improve with more aggressive treatment such as transfer to the intensive care unit, close monitoring, and early transjugular intrahepatic portosystemic shunting.

The aim of this study was to compare the performance of Child, MELD, Rockall, Blatchford, and AIMS65 scores as predictors of 6-week rebleeding and mortality in patients with AVB.

## METHODS

A retrospective study was conducted at the State University of Campinas Hospital between January 2016 and July 2019. All patients with suspected UGIB, presenting with a clinical history of hematemesis, coffee-ground emesis, or melena were hemodynamically stabilized, received omeprazole (80 mg) and/or octreotide (intravenous bolus of 50 μc), according to their medical history, and underwent endoscopic examination.

Patients with AVBs were included in this study. They were considered to present with variceal bleeding when active or recent bleeding stigmata of gastroesophageal varices were identified, if varices were diagnosed in the context of a recent UGIB with no other bleeding source or if there were ulcers from previous banding ligation.

After confirming the variceal etiology, all patients received a splanchnic vasoconstrictor (octreotide), an initial dose intravenous bolus of octreotide (50 μc; if not previously received) and subsequently maintained at 50 μc per hour until hospital discharge or for up to five days.

Antibiotic prophylaxis was performed with a quinolone (ciprofloxacin; 500 mg orally, every 12h) or third-generation cephalosporin (intravenous ceftriaxone 1g, once daily).

Data from each patient were collected in a standardized form containing clinical and laboratory data, score calculations, and endoscopic findings. These forms were placed in a database and used for outpatient follow-up.

### Clinical outcomes

The primary outcome was 6-week rebleeding and was defined as clinical evidence of bleeding (presence of hematemesis and/or melena), with either shock or a decrease in hemoglobin concentration of at least 2 g/dL over 24 hours.

The secondary outcome was 6-week mortality. Death was defined as all-cause mortality until the follow-up period and was determined through hospital medical records.

### Statistical analysis

Statistical analysis was performed using the SAS software for Windows (version 9.4; SAS Institute Inc., Cary, NC, USA). A chi-square or Fisher's exact test were performed for comparisons between categorical variables, as appropriate. A Mann-Whitney test was performed to compare numerical variables. Receiver operating characteristic (ROC) curves were created to define the cutoff points of the scores concerning the outcomes.

The discriminative abilities of the scoring systems to predict 6-week rebleeding and death were evaluated using ROCs with 95% confidence intervals (CI). An area under the ROC (AUROC) of 0.5 indicated no predictive power, whereas AUROCs of 1-0.9, 0.9-0.8, and 0.8,0.7 indicated excellent, good, and acceptable predictive power, respectively.

### Ethics

This study was approved by the State University of Campinas School of Medical Sciences Ethics Committee (CAAE 71177817.3.0000.5404).

## RESULTS

Overall, 247 patients with AVB were admitted to our hospital. Twenty-five patients with incomplete records were excluded, and 222 patients were studied (mean age: 56.15±13 years; range, 19-84 years; 77% male). The clinical characteristics of patients with AVB are summarized in Table 1.

Among the 222 patients, the presenting symptoms were hematemesis (78.4%), melena (66.2%), and hematochezia (4.5%). Previous AVB was reported in 96 (44.4%) patients, and 72 (32.4%) were on pharmacological and/or endoscopic prophylaxis.

The main cause of portal hypertension was liver cirrhosis, which was diagnosed in 182 (82%) patients, with alcohol (n=79, 43.4%) being the primary etiology.

Regarding liver severity scores, the mean (range) Child score was 8±2.2 (2-14) and patients were classified as Child A (n=65, 31.7%), B (n=86, 42%), and C (n=54, 26.3%). The mean (range) MELD score was 15.3±6.6 (6-45).

Bleeding was related to esophageal varices in 194 (87.4%) patients, gastric varices in 26 (11.7%) patients, and ulcers from previous banding ligation in 2 (0.9%) patients. Endoscopic treatment was performed in 203 (91.4%) patients [endoscopic band ligation (90%), cyanoacrylate (9.3%), and ethanolamine injection (0.4%)]. Initial success of endoscopic treatment was achieved in 96% of cases. In three patients with massive bleeding without initial hemostasis, balloon tamponade was necessary.

Ultrasonography was performed on 179 patients. Of these, portal vein thrombosis was diagnosed in 12 (6.7%) patients and hepatocellular carcinoma in 26 (14.5%) patients.

The primary outcome was a 6-week rebleeding rate of 14% (n=31). Among these, 27 patients had liver cirrhosis [Child A (n=5, 18.5%), Child B (n=9, 33.3%), and Child C (n=13, 48.2%), *p*=0.04]. Of the 31 patients, 10 rebled in the first five days.

Regarding the secondary outcome, the 6-week mortality rate was 18.5% (n=41). Of these 41 patients, 37 had liver cirrhosis. According to Child scores, 2 (5.4%) patients were classified as Child A, 9 (24.3%) patients as Child B, and 25 (67.5%) patients as Child C (*p*≤0.0001). Of the 41 patients, 13 died during the first five days.

Comparing patients with or without liver cirrhosis, there were no differences in 6-week rebleeding (14.8% *vs.* 10%, *p*=0.61) and mortality (20.3% *vs.* 10%, *p*=0.17), respectively.

The cause of death was infection in 22 (53.6%) patients, hypovolemic shock in 14 (34.2%) patients, renal failure in 2 (4.8%) patients, and other causes in 3 (7.3%) patients. Table 2 displays the clinical outcomes of patients with AVB.

### Predictive performance of clinical scores for 6-week rebleeding

There were no differences in Rockall (4.8±2 *vs.* 4.4±1.9, *p*=0.15) and Blatchford (13.2±3 *vs.* 12.1±3.5, *p*=0.13) scores between patients with and without rebleeding. Neither Rockall (AUROC=0.59; 95% CI=0.48-0.69) nor Blatchford (AUROC=0.57; 95% CI=0.49-0.69) scores were helpful in identifying patients at a higher risk of rebleeding.

The mean MELD scores were 17.1±8.9 and 15±6.2 in rebleeding and non-rebleeding patients, respectively (*p*=0.40). The AUROC was 0.56 (95% CI=0.43-0.64), indicating that this score did not help identify patients at higher risk of rebleeding. 

The AIMS65 (2.2±1.2 *vs.* 1.5±1.2, *p*=0.006) and Child (8.9±2.2 *vs.* 7.8±2.2, *p*=0.01) scores were significantly higher in rebleeding patients. However, the AUROCs were 0.61 (95% CI=0.55-0.74) and 0.63 (95% CI=0.53-0.74), respectively, indicating that these scores did not help identify patients at risk for rebleeding.

**Figure 1:**
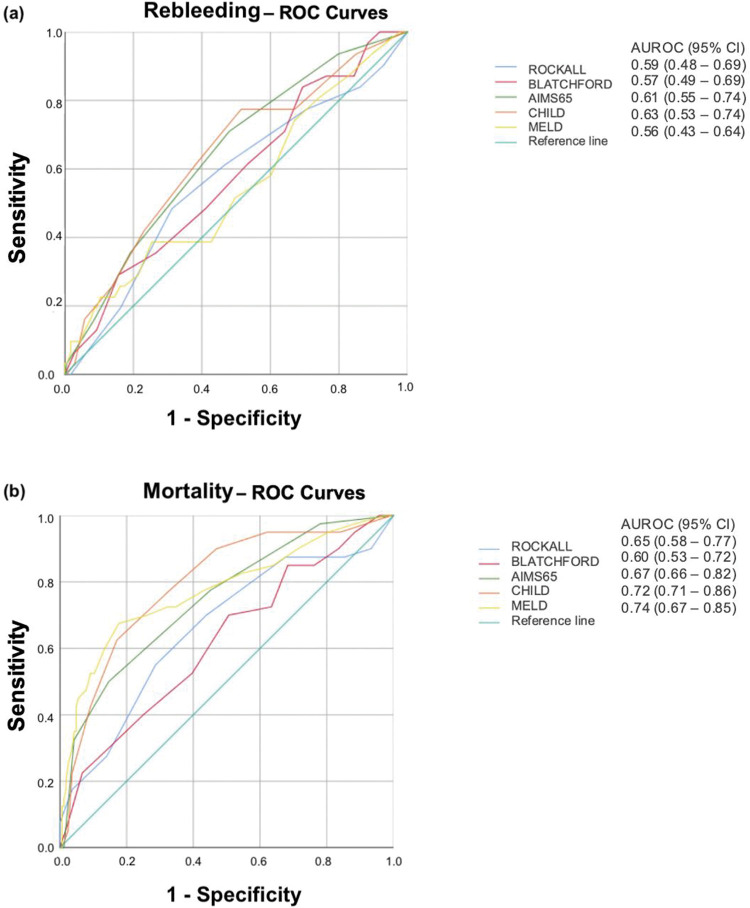
Receiver-operating characteristic curves (ROC) for Rockall, Glasgow-Blatchford, AIMS65, Child-Pugh, and MELD scores in predicting (a) rebleeding and (b) death in patients with acute variceal bleeding.

The ROCs for rebleeding prediction are shown in Figure 1a.

### Predicting mortality

All three nonvariceal scores were significantly higher in patients who died than in survivors (Rockall: 5.4±2.1 *vs.* 4.2±1.8, *p*=0.0004; Blatchford: 13.4±3.1 *vs.* 11.9±3.4, *p*=0.0147; AIMS65: 2.6±1.2 *vs.* 1.4±1.1, *p*≤0.0001). However, Rockall (AUROC=0.65; 95% CI=0.58-0.77), Blatchford (AUROC=0.60; 95% CI =0.53-0.72), and AIMS65 (AUROC=0.67; 95% CI=0.66-0.82) scores were not useful for predicting mortality (Figure 1b).

Liver risk scores were also significantly higher in patients who died than in survivors (Child: 9.9±1.9 *vs.* 7.5±2.0, *p*<0.0001 and MELD: 21.3±8.1 *vs.* 13.9±5.4, *p*<0.0001). These scores were acceptable for predicting mortality (AUROC=0.72, 95% CI=0.71-0.86; and AUROC=0.74, 95% CI=0.67-0.85, respectively; Figure 1b). The cutoffs for Child and MELD scores were 9 (sensitivity, 0.83; specificity, 0.72) and 17 (sensitivity: 0.67; specificity: 0.82), respectively.

## DISCUSSION

Our results showed that Rockall, Blatchford, AIMS65, Child, and MELD scores were not helpful in identifying patients at a higher risk of 6-week rebleeding (AUROC<0.7).

According to the literature, the factors associated with the highest risk of rebleeding are Child B status with active bleeding, Child C status, MELD score >18, and pressure gradient in the hepatic vein ≥20 mmHg (6,18,19).

None of the scores considered the severity of portal hypertension and/or presence of portal vein thrombosis. Although the presence of liver disease is considered in Rockall and Blatchford scores, many patients may be underscored because of unknown hepatic disease.

Other authors have demonstrated that nonvariceal UGIB scores were not accurate in assessing rebleeding in AVB, limiting their potential for routine use (15,16).

We evaluated 6-week rebleeding. Rockall, Blatchford, and AIMS65 scores consider variables related to the immediate systemic impact of bleeding (admission systolic blood pressure, heart rate, hemoglobin, and blood urea nitrogen) and may correlate with short-term outcomes but not with long-term complications. Some authors have demonstrated the usefulness of these scores for predicting in-hospital adverse outcomes in patients with AVB (17,20). However, there are conflicting studies on the use of these scores for AVB (16,21).

In our study, Child and MELD scores performed best in predicting 6-week mortality (AUROC=0.72 and 0.74, respectively), but not for predicting rebleeding. Robertson et al. also reported that liver disease severity scores were poor predictors of rebleeding and failed to reach statistical significance (20).

Child and MELD scores are associated with the severity of liver disease. In our series, most patients who died had liver dysfunction (24.3% were classified as Child B and 67.5% were classified as Child C, *p*<0.0001), in addition to higher MELD scores (21.3±8.1 *vs.* 13.9±5.4, *p*<0.0001).

The main cause of death in our study was infection (53.6%), despite prophylactic antibiotic therapy, followed by gastrointestinal bleeding (34.2%). Patients with AVB are at a higher risk of developing infections and, consequently, worsening liver function (22). Additionally, advanced cirrhosis is a risk factor for bacterial translocation (increased intestinal permeability, impaired reticuloendothelial function, and decreased complement factor synthesis, allowing passage of bacteria and bacterial components of intestinal origin into the bloodstream, predisposing to infection). Infection can increase portal hypertension due to increased vasoconstrictor production, initiating a vicious cycle (23).

In a previous study carried out on our institution from March 2010 to April 2013, among 164 patients with AVB, MELD scores >15 were shown to be strongly associated with death. In this series, 70.7% of patients who died had MELD scores >15 (24). Another study demonstrated that MELD scores ≥19 predicted mortality rate of ≥20%, whereas scores of <11 predicted a mortality rate of <5% (25).

Our study has some limitations. This was a retrospective study conducted in a single center with extensive experience in the management of AVB. Additionally, our institution is a tertiary university hospital, acting as a referral center for five million inhabitants and includes a liver transplantation unit. Selection bias is unavoidable, and about 70% of the studied patients had hepatic dysfunction.

A major strength of our study is the duration of follow-up (6 weeks). In the Baveno VI consensus, 6 weeks was suggested as the primary endpoint for AVB studies. Adverse outcomes can be underestimated in short-term studies (usually in-hospital or 5-day studies). In the present study, there were greater incidences of rebleeding and mortality during the 6-week interval than during the 5-day interval (Table 2).

Further studies are necessary to assess the effectiveness of applying these scores in stratifying the risk of AVB, which remains life-threatening in patients with portal hypertension, as rapid prognostic assessment can further improve its management.

In conclusion, non-variceal scores (Rockall, Blatchford, and AIMS65) are not useful in predicting 6-week rebleeding or mortality in patients with AVB. Child and MELD scores can discriminate patients at higher risk of 6-week mortality.

## AUTHOR CONTRIBUTIONS

Aluizio CLS and Nagasako CK were responsible for the study conception, planning, analysis, interpretation of data, manuscript writing and review for critical intellectual content, and approval of the final manuscript version for publication. Montes CG and Reis GFSR were responsible for the study conception, planning, analysis, and interpretation of data.

## Figures and Tables

**Table 1:** Clinical characteristics of patients with acute variceal bleeding.

Variables	All patients (n=222)
Age	56.15±13
Male sex	174 (77)
Active drinking, n=202	52 (25.7)
Etiology of Portal Hypertension	
Cirrhosis	182 (82)
Schistosomiasis	8 (3.6)
Thrombosis	2 (0.9)
Budd-Chiari	2 (0.9)
Post Liver Transplantation	1 (0.45)
Unknown	27 (12.2)
Child	8±2.2
MELD	15.3±6.6
Hematemesis	174 (78.4)
Heart Rate (bpm)	93.5±16.2
Systolic Blood Pressure (mmHg)	113.3±20.5
Thrombosis, n=179	12 (6.7)
Hepatocellular Carcinoma, n=179	26 (14.5)
Hemoglobin (g/dL)	8.7±2.3
Bilirubin (mg/dL)	2.6±3.7
Albumin (g/dL)	2.7±0.7
INR	1.5±0.5

Values represent means±standard deviation or n (%).

**Table 2:** Clinical outcomes of patients with acute variceal bleeding.

Variables	All patients (n=222)
Blood Transfusion	90 (40.6)
Site of Bleeding	
Esophageal Varices	194 (87.4)
Gastric Varices	26 (11.7)
Ulcer from Previous Banding	2 (0.9)
Stigma of recent hemorrhage	208 (93.7)
Endoscopic Treatment	203 (91.5)
Use of balloon tamponade	3 (0.01)
Rebleeding	
5-day	10 (4.5)
6-week	41 (18.5)
Death	
5-day	13 (5)
6-week	41 (18.5)
Causes of death	
Infection	22 (53.6)
Hypovolemic shock	14 (34.2)

Values represent means±standard deviation or n (%).
